# Supplementary material for: Resolving the full spectrum of human genome variation using Linked-Reads
Source: Genome Res. 2019 Apr;29(4):635–45. doi: 10.1101/gr.234443.118 (PMC6442396; doi:10.1101/gr.234443.118)
Supplement: Supplemental Material [file supp_gr.234443.118_Supplemental_longranger-2.2.zip › longranger-2.2.tar.bz2!/lr2.2-public/lib/stan/cmdstan/stan/doc/api/html/arg__tolerance_8hpp_source.html]

Stan: src/stan/gm/arguments/arg\_tolerance.hpp Source File


|  |  |
| --- | --- |
|  | Stan  2.5.0  probability, sampling & optimization |


- Main Page
- Related Pages
- Namespaces
- Classes
- Files

- File List
- File Members

All Classes Namespaces Files Functions Variables Typedefs Enumerations Enumerator Friends Macros Pages

- src
- stan
- gm
- arguments

arg\_tolerance.hpp

Go to the documentation of this file.

1 #ifndef STAN\_\_GM\_\_ARGUMENTS\_\_TOLERANCE\_HPP

2 #define STAN\_\_GM\_\_ARGUMENTS\_\_TOLERANCE\_HPP

3

4 #include <boost/lexical\_cast.hpp>

5

6 #include <stan/gm/arguments/singleton\_argument.hpp>

7

8 namespace stan {

9

10  namespace gm {

11

12  class arg\_tolerance : public real\_argument {

13

14  public:

15

16  arg\_tolerance(const char \*name, const char \*desc, double def) : real\_argument() {

17  \_name = name;

18  \_description = desc;

19  \_validity = "0 <= tol";

20  \_default = boost::lexical\_cast<std::string>(def);

21  \_default\_value = def;

22  \_constrained = true;

23  \_good\_value = 1.0;

24  \_bad\_value = -1.0;

25  \_value = \_default\_value;

26  };

27

28  bool is\_valid(double value) { return value >= 0; }

29

30  };

31

32  } // gm

33

34 } // stan

35

36 #endif

stan::gm::arg\_tolerance::is\_valid

bool is\_valid(double value)

**Definition:** arg\_tolerance.hpp:28

stan::gm::singleton\_argument::\_validity

std::string \_validity

**Definition:** singleton\_argument.hpp:151

stan::gm::arg\_tolerance

**Definition:** arg\_tolerance.hpp:12

stan::gm::singleton\_argument::\_value

T \_value

**Definition:** singleton\_argument.hpp:154

stan::gm::valued\_argument::\_default

std::string \_default

**Definition:** valued\_argument.hpp:47

stan::gm::singleton\_argument

**Definition:** singleton\_argument.hpp:44

stan::gm::singleton\_argument::\_bad\_value

T \_bad\_value

**Definition:** singleton\_argument.hpp:160

stan::gm::singleton\_argument::value

T value()

**Definition:** singleton\_argument.hpp:124

stan::gm::argument::\_name

std::string \_name

**Definition:** argument.hpp:76

stan::gm::singleton\_argument::\_constrained

bool \_constrained

**Definition:** singleton\_argument.hpp:157

stan::gm::argument::name

std::string name() const

**Definition:** argument.hpp:26

stan::gm::arg\_tolerance::arg\_tolerance

arg\_tolerance(const char \*name, const char \*desc, double def)

**Definition:** arg\_tolerance.hpp:16

stan::gm::singleton\_argument::\_default\_value

T \_default\_value

**Definition:** singleton\_argument.hpp:155

singleton\_argument.hpp

stan::gm::singleton\_argument::\_good\_value

T \_good\_value

**Definition:** singleton\_argument.hpp:159

stan::gm::argument::\_description

std::string \_description

**Definition:** argument.hpp:77


---

[ Stan Home Page ]

*© 2011–2014,
Stan Development Team.*
